# Impact of the COVID‐19 pandemic on the mortality among patients with colorectal cancer in Hiroshima, Japan: A large cancer registry study

**DOI:** 10.1002/cam4.6630

**Published:** 2023-10-25

**Authors:** Daisuke Miyamori, Tsukasa Kamitani, Shuhei Yoshida, Yuya Shigenobu, Kotaro Ikeda, Yuka Kikuchi, Saori Kashima, Yosuke Yamamoto

**Affiliations:** ^1^ Department of General Internal Medicine Hiroshima University Hospital Hiroshima Japan; ^2^ Section of Education for Clinical Research Kyoto University Hospital Kyoto Japan; ^3^ Graduate School for International Development and Cooperation Hiroshima University Hiroshima Japan; ^4^ Environmental Health Sciences Laboratory, Graduate School of Advanced Science and Engineering Hiroshima University Hiroshima Japan; ^5^ Department of Healthcare Epidemiology, School of Public Health in the Graduate School of Medicine Kyoto University Kyoto Japan

**Keywords:** colorectal cancer, COVID‐19 pandemic, mortality, overall survival

## Abstract

**Background:**

This retrospective cohort study aimed to evaluate the impact of the COVID‐19 pandemic on colorectal cancer care and mortality using a large cancer registry in Hiroshima Prefecture, Japan. The study aimed to estimate the all‐cause mortality rates within 1 year of diagnosis among colorectal cancer patients diagnosed during the pandemic period (2020 and 2021) compared to those diagnosed during the pre‐pandemic period (2018 and 2019).

**Methods:**

The day of diagnosis was set as Day 0 and Cox regression models were utilized to estimate crude hazard ratios (HRs) and adjusted HRs, accounting for age, sex, cancer stage, and treatment status. Two sensitivity analyses of overall survival were performed with different cutoffs of the pre‐pandemic/pandemic periods and year‐to‐year comparisons. Subgroup analyses were performed using likelihood ratio tests.

**Results:**

A total of 15,085 colorectal cancer patients were included, with 6499 eligible for follow‐up. A median age of included patients was 72 years old, of which 59% were male. The distribution of cancer stages showed little variation between the pre‐pandemic and pandemic periods. With a median follow‐up of 177 days, the number of events was 316/3111 (173 events per 1000 person‐years [E/1000PY], 95% confidence interval [CI]: 154–192 E/1000PY) in the pre‐pandemic period, and 326/2746 (245 E/1000PY, 95% CI: 220–274 E/1000PY) in the pandemic period (crude HR: 1.42, 95% CI: 1.22–1.66; adjusted HR: 1.25, 95% CI: 1.07–1.46). The two sensitivity analyses and subgroup analyses consistently supported these findings.

**Conclusions:**

The study revealed an increased colorectal cancer mortality during the pandemic period, suggesting a continuous impact of the COVID‐19 pandemic on the known and unknown risk factors for colorectal cancer for several years. Further studies are necessary to mitigate the adverse effects on patient outcomes.

## Graphical abstract

This study first describes the mortality risk of colorectal cancer diagnosed during the COVID‐19 pandemic. Colorectal cancer patients diagnosed during the pandemic were 1.4 times higher mortality risk than those during pre‐pandemic. The prognostic impact of delaying diagnosis and treatment may persist in 2020 and 2021.

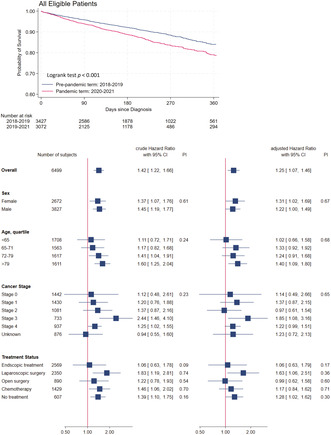

## 1 BACKGROUND

COVID‐19 has been a significant public health problem since its recognition December 2019 at Wuhan, China. The COVID‐19 pandemic has been associated with delays in new cancer diagnoses, diagnosis at more advanced stages, and delays in initiating effective treatment.^1^, ^2^, ^3^, ^4^, ^5^, ^6^

In January 2020, the first case of COVID‐19 was diagnosed in Japan, and the Cabinet Office declared the State of Emergency in April 2020.^7^ The Ministry of Health, Labor, and Welfare proposed postponing cancer screening for 1 year starting in April 2020, and withdrawn immediately thereafter due to receiving objections to the postponement of cancer screening. However, during first wave of COVID‐19 (March to May 2020), the number of colorectal cancer screening decreased 50% than that of before pandemic.^8^ As in other Organization for Economic Cooperation and Development countries, the number of upper and lower gastrointestinal endoscopies performed in medical institutions in Japan decreased by 30%–100% during the COVID‐19 epidemic compared with those in previous years.^3^, ^9^, ^10^, ^11^

In addition, cancer care and treatment changed, with treatment algorithms that differed from standard practice.^12^, ^13^ Delays in the time to starting chemotherapy and radiation therapy also occurred.^14^ A survey of the number of diagnosed colorectal cancers at two Japanese hospitals showed a 13% decrease in the overall number of colorectal cancers diagnosed during 2020 compared with 2019, with a decrease of approximately 30% in the number of Stage 0 and 1 cancers that were diagnosed.^2^

The assessment of colorectal cancer, including histology by colonoscopy, is essential, especially in the early stages of the disease. Although the COVID‐19 pandemic resulted in isolation and a reluctance to seek medical care, few studies have examined the impact of the COVID‐19 pandemic on the diagnosis and prognosis of colorectal cancer using large databases.^15^ Considering the slow progression from colorectal polyps to carcinoma,^16^ the impact of the COVID‐19 pandemic in terms of delayed diagnosis, treatment, and quality of care might not appear rapidly and needs to be evaluated continuously.

In this study, we compared the baseline characteristics of patients with newly diagnosed colorectal cancer during the pre‐pandemic and pandemic periods and the mortality risk after diagnosis in Hiroshima, Japan, using a large cancer registry.

## 2 METHODS

### 2.1 Study design and setting

This was a retrospective cohort study performed in Hiroshima Prefecture, Japan, which has a population of 2.81 million using cancer registry data.^17^ In Hiroshima Prefecture, data are collected by the Hiroshima Cancer Medicine Collaboration Council Institutional Cancer Registration Subcommittee, mainly at hospitals designated for cancer treatment.^18^, ^19^ This database includes demographic, staging, type of treatment, and mortality data for patients with newly diagnosed cancer. The database consists of 15 institutions, including one university hospital, one cancer hospital, and 13 cancer‐designated hospitals, all of which provide standardized cancer care. The registration rate of colorectal cancer was approximately 77% of the colorectal cancer registered in the National Cancer Registry of Hiroshima Prefecture.^20^ Records on each hospital were extracted from medical record information by qualified medical information personnel. We extracted data on colorectal cancer diagnosed between 2018 and 2021 from this database.

### 2.2 Inclusion criteria

All patients with newly diagnosed cancer during the study period whose ICD‐O‐3 site code indicated colorectal cancer (Table S1) were eligible for the study.^21^

### 2.3 Assessment of the primary endpoint

The primary endpoint was the occurrence of mortality from any cause, since the reason for death was not clear from the cancer registry. The follow‐up period was based on patient medical records at each hospital, which were extracted and used for the study. As cases diagnosed in 2021 only have a follow‐up period until the end of 2022, the maximum follow‐up period was set at 1 year for all cases. Cases were censored if the follow‐up period ended during the study period. Patients who were transferred between institutions were followed continuously, provided that they were transferred between the 15 study institutions.

### 2.4 Definition of the pandemic period

In Japan, repeated outbreaks of COVID‐19 occurred throughout the 2020 and 2021 (Figure S1A). Unlike many other countries, Japan did not implement a lockdown. Instead, the government issued restrictions on activities through infection prevention measures and limited non‐essential outings until April 2023. The political decision for postponed cancer screening was carried out in April 2020 and withdrawn 2 days later. In this study, 2020 and 2021 were used as the pandemic period, and the management of colorectal cancer diagnosed during 2020 and 2021 was compared with that of colorectal cancer diagnosed in 2018 and 2019 (pre‐pandemic period).

### 2.5 Covariates

Data on age, sex, cancer stage, and treatment status were extracted. For the cancer stage, patients were registered based on the Union for International Cancer Control, eighth edition.^22^ We used the data of the clinical cancer stage because pathological stages were confirmed only for patients who received surgical treatment. Treatment status was also assessed by recording the presence or absence of endoscopic therapy, laparoscopic resection, open surgery, chemotherapy, radiation therapy, and endocrine therapy, as well as the interval from the day of diagnosis to starting treatment at each hospital. The process of cancer detection was classified as abnormal cancer screening, follow‐up for other colorectal diseases, detection in autopsy, examination because of symptoms, and other reasons.

### 2.6 Statistical analysis

Analyses were conducted using Stata version 17 MP (StataCorp LLC, College Station, TX, USA). First, we described the patient characteristics according to whether the cancer was diagnosed during the pandemic period (in 2020 and 2021) or the pre‐pandemic period (in 2018 and 2019). A two‐sided *p*‐value of less than 0.05 was interpreted as statistically significant.

Survival analysis was conducted. The date of diagnosis was set as Day 0 in the analysis. In this study, we defined censoring as any patient who was lost to follow‐up due to a lack of medical records or who was still alive at the end of the 1‐year study period. All‐cause mortality was set as a censoring event. Kaplan–Meier curves were plotted for overall mortality, and the pandemic and pre‐pandemic periods were compared using the log‐rank test. Cox proportional hazards regression was utilized to estimate crude and adjusted HRs for the main analysis. The adjusted HRs were adjusted for sex, age, cancer stage, and treatment status. We assessed the assumption of proportional hazards for Cox regression analysis by examining the Schoenfeld residuals over time. Subgroup analyses were conducted for each category of sex, age, cancer stage, and treatment status, and the likelihood ratio test was utilized to assess whether adding interaction terms between the pandemic period and each subgroup affected the goodness of fit of the original models.

In addition, we conducted two sensitivity analyses with different exposure periods: (1) setting the pandemic period from July 2019 to December 2021, and the pre‐pandemic period from January 2018 to June 2019, because colorectal cancer cases diagnosed in late 2019 were considered to have a higher probability of receiving cancer care during the COVID‐19 pandemic; and (2) a year‐to‐year comparison with 2018 was used as the reference period.

## 3 RESULTS

**FIGURE 1:**
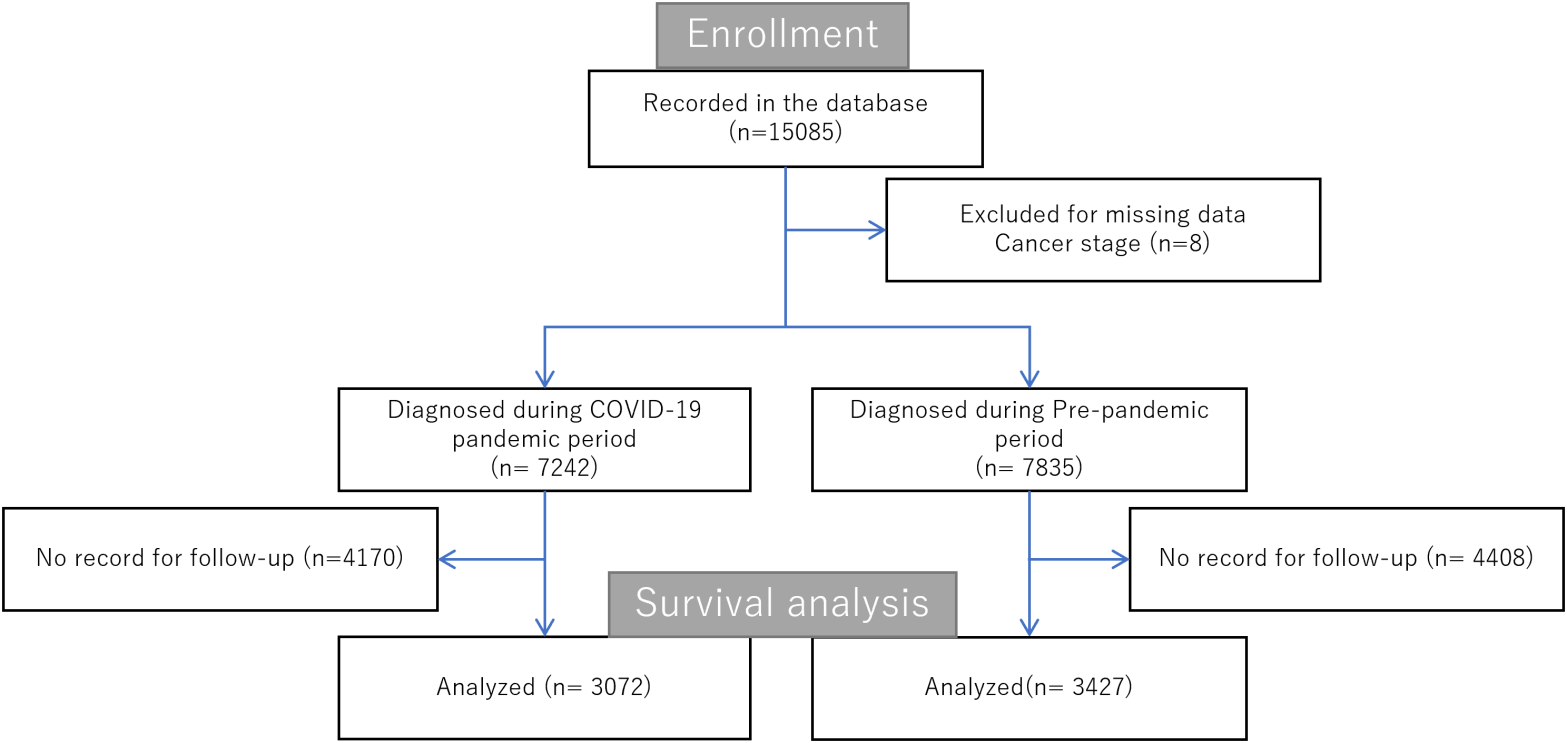
Flow chart. A total of 15,085 patients were assessed for eligibility; 10 patients were excluded for because of missing covariates. Among the 7242 and 7835 patients diagnosed with colorectal cancer during the pandemic and pre‐pandemic periods, and 3072 and 3427 patients, respectively, were included in the main analysis.

Figure 1 shows the flow chart of the patients included in the study. A total of 15,085 participants met the inclusion criteria. After excluding patients with missing covariates, 7242 and 7843 were diagnosed with colorectal cancer during the pandemic and pre‐pandemic periods, respectively. Survival analysis was conducted using data on the 6802 individuals who were traceable.

Baseline characteristics (Table 1) showed a median (IQR) age of 72 years (64–79 years) and male predominance (59%). The cancer stage was 22%, 22%, 17%, 11%, and 14%, from Stages 0 to 4, respectively, and the stage was unknown in 13%. The stage at diagnosis did not vary between the pandemic and pre‐pandemic periods. Endoscopic treatment was performed in approximately 40% of the patients, and laparoscopy and open surgery were used in 36% and 14% of patients, respectively. The proportion of patients without receiving any treatment was 9% and did not vary between the pandemic and pre‐pandemic periods. The results of the comparisons of other factors are shown in Table S2, and the characteristics of all the patients enrolled in this study are shown in Table S3.

**TABLE 1:** Baseline characteristics of the patients.

	Total	Pre‐pandemic	Pandemic
	*N* = 6499	*N* = 3427	*N* = 3072
Age, Median (IQR)‐year	72 (64–79)	72 (64–79)	72 (65–79)
Male sex, No (%)	3827 (59%)	2029 (59%)	1798 (59%)
Cancer stage, No (%)			
0	1442 (22%)	809 (24%)	633 (21%)
1	1430 (22%)	746 (22%)	684 (22%)
2	1081 (17%)	575 (17%)	506 (16%)
3	733 (11%)	416 (12%)	317 (10%)
4	937 (14%)	470 (14%)	467 (15%)
Unknown	876 (13%)	411 (12%)	465 (15%)
Treatment status, No (%)			
Endoscopic treatment	2569 (40%)	1332 (39%)	1237 (40%)
Laparoscopic surgery	2350 (36%)	1183 (35%)	1167 (38%)
Open surgery	890 (14%)	580 (17%)	310 (10%)
Radiotherapy	152 (2%)	80 (2%)	72 (2%)
Chemotherapy	1429 (22%)	803 (23%)	626 (20%)
Endocrine therapy	1 (0%)	0 (0%)	1 (0%)
No treatment recorded	607 (9%)	298 (9%)	309 (10%)
Time from diagnosis (days), median (IQR)			
Endoscopic treatment	0.0 (0.0–25.0)	0.0 (0.0–27.0)	0.0 (0.0–21.0)
Laparoscopic surgery	30.0 (19.0–49.0)	34.0 (20.0–53.0)	28.0 (17.0–44.0)
Open surgery	22.0 (10.0–38.0)	23.0 (11.0–38.5)	20.0 (8.0–38.0)
Chemotherapy	26.0 (14.0–37.0)	26.0 (15.5–39.0)	22.0 (13.0–34.5)
Radiotherapy	54.0 (30.0–71.0)	56.0 (31.0–73.0)	51.0 (29.0–69.0)
Endocrine therapy	55.0 (55.0–55.0)	N/A	55.0 (55.0–55.0)

*Note*: Cancer stage was classified based on the classification eighth edition of TNM classification of Malignant Tumors, by Union for International Cancer Control. Treatment status were based on the report from each hospital. The category of no treatment group was set if the patients had not received any of endoscopic, laparoscopic, surgical resection, chemotherapy, radiation therapy, and endocrine therapy.

Abbreviations: IQR, interquartile range, No, number.

**FIGURE 2:**
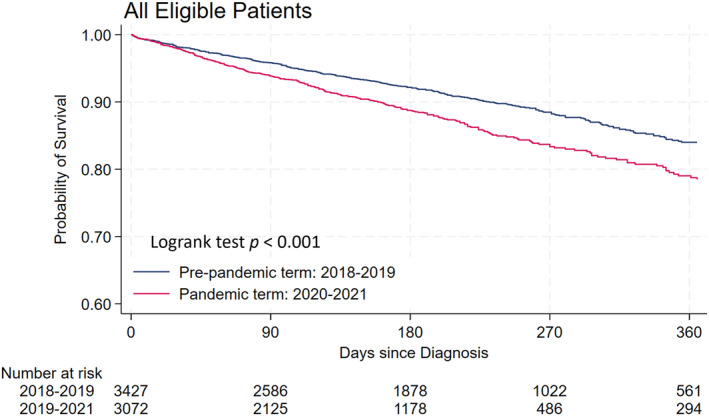
Kaplan–Meier curves of overall survival among patients with colorectal cancer during the pre‐pandemic and pandemic periods. Kaplan–Meier curves show the estimate of the probability of survival from all‐cause deaths among patients with colorectal cancer.

The Kaplan–Meier analysis to estimate the overall survival of patients with colorectal cancer is shown in Figure 2. The median observation period was 177 days. The Kaplan–Meier curve showed that the estimated overall survival rate at 1 year was 81.2%. The number of events was 316/3111 (173 events per 1000 person‐years [E/1000PY], 95% confidence interval [CI]: 154–192 E/1000PY) in the pre‐pandemic period, and 326/2746 (245 E/1000PY, 95% CI: 220–274 E/1000PY) in the pandemic period. Table 2 shows the crude HRs and adjusted HRs of the Cox proportional hazards regression model for the main and two sensitivity analyses. In the main analysis, the crude HR and HR adjusted for age, sex, cancer stage, and treatment status were 1.42 (95% CI: 1.22–1.66) and 1.25 (95% CI: 1.07–1.46), respectively, during the pandemic period compared with the pre‐pandemic period. The HR for the pandemic period on colorectal cancer mortality approached null after adjustment with covariates.

**TABLE 2:** Crude and adjusted HR from all‐cause mortality among patients with colorectal cancer, according to different cutoff between pandemic and pre‐pandemic periods.

Model	Period	Crude HR	95% CI	Adjusted HR^a^	95% CI
Main analysis	2018–2019	Ref		Ref	
	2020–2021	1.42	1.22, 1.66	1.25	1.07, 1.46
Sensitivity analysis 1	2018–early 2019	Ref		Ref	
	Late 2019–2021	1.43	1.22, 1.69	1.36	1.16, 1.60
Sensitivity analysis 2	2018	Ref		Ref	
	2019	1.22	0.98, 1.52	1.36	1.09, 1.70
	2020	1.58	1.27, 1.98	1.55	1.24, 1.94
	2021	1.57	1.25, 1.97	1.38	1.10, 1.73

*Note*: The mortality risk of colorectal cancer during pandemic compared to pre‐pandemic was analyzed for 3 different cutoff of pandemic and pre‐pandemic period. The reference period was set as from January 2018 to December 2019, January 2018 to September 2019, and 2018, for main, sensitivity 1 and sensitivity 2 analyses, respectively. The Schoenfeld residual test showed no violation of the proportional hazards assumption between the pandemic and pre‐pandemic periods for main (*p* = 0.28), sensitivity 1 (*p* = 0.21), and sensitivity 2 (*p* = 0.41) analyses.

Abbreviations: CI, confidence interval; HR, hazard ratio; Ref, reference.

^a^ Adjusted for age, sex, cancer stage, and treatment status.

The results of the two sensitivity analyses exploring the impact of different cutoffs for defining the pre‐pandemic and pandemic periods on the association between the COVID‐19 pandemic and colorectal cancer mortality risk are shown in Table 2 and Figures S2 and S3. When the cutoff was changed to June 2019 to account for potential decreased access to treatment of patients diagnosed with colorectal cancer in the second half of 2019, the crude HR was 1.43 (95% CI: 1.19–1.62) and the adjusted HR was 1.36 (95% CI: 1.16–1.60). In the second sensitivity analysis, which compared survival in patients diagnosed in each year of 2019, 2020, and 2021 to the reference year of 2018, the mortality risk of patients diagnosed in 2020 and 2021 was significantly higher than that in 2018 (2020, crude HR: 1.58, 95% CI: 1.27–1.98; 2021, crude HR: 1.57, 95% CI: 1.25–1.97). There was no violation of the proportional hazard assumptions in the main analysis or either of the two sensitivity analyses.

**FIGURE 3:**
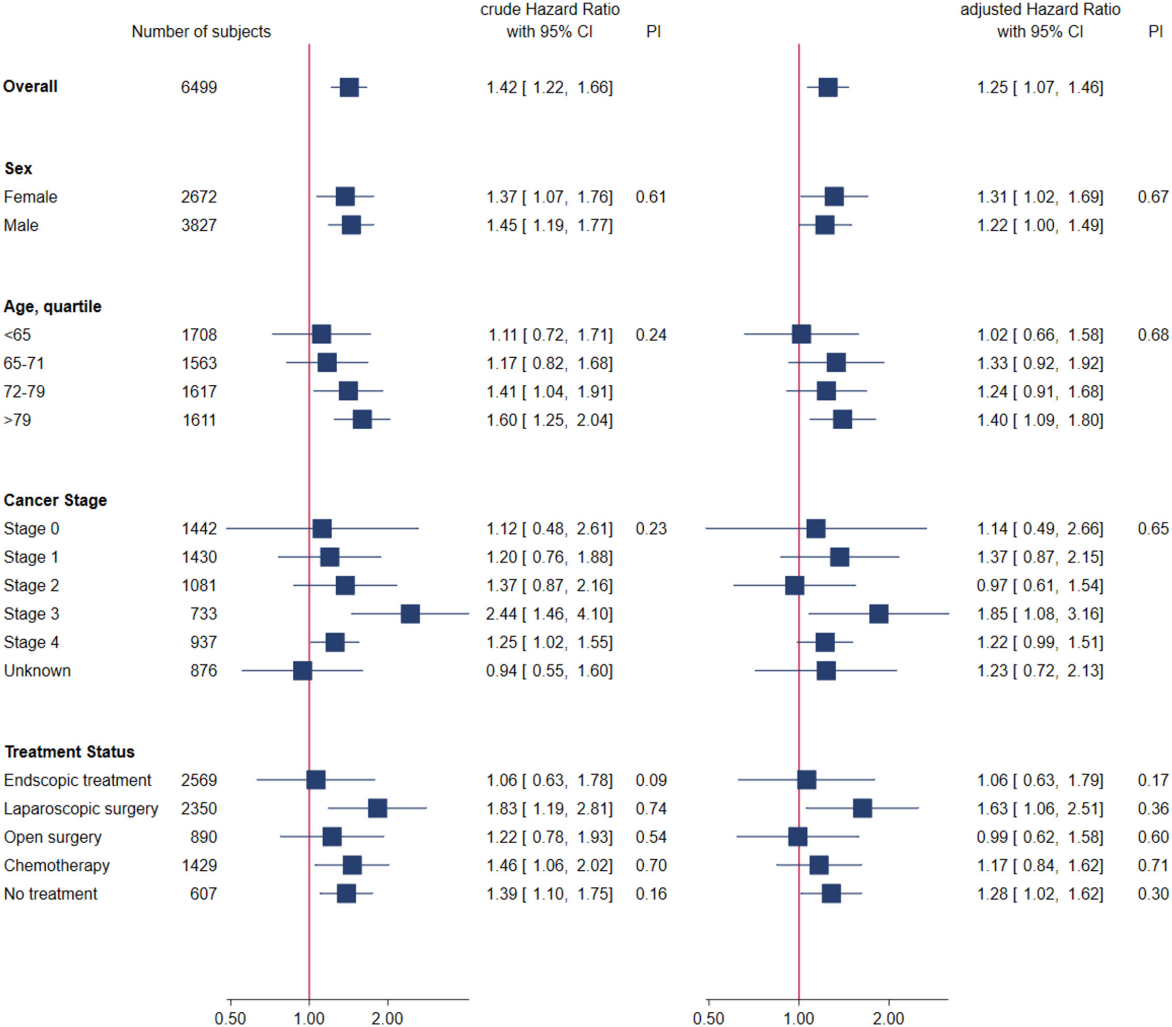
Crude and adjusted subgroup analyses of the hazard ratios for overall risk of death within 1 year of diagnosis in patients with colorectal cancer diagnosed during the COVID‐19 pandemic period relative to the pre‐pandemic period. The solid vertical line indicates hazard ratio of 1. Age was categorized into quartiles. The forest plot on the left shows the crude HRs, and the forest plot on the right shows the adjusted HRs. The interaction between pandemic status and each category was analyzed using the likelihood ratio test. Each adjusted hazard ratio was adjusted for all other variables. CI, confidence interval, HR, hazard ratio.

Subgroup analyses were performed to evaluate the potential interaction between the pandemic period and colorectal cancer mortality risk (Figure 3). The subgroups were based on age, sex, cancer stage, and treatment status. The results showed that the trend of an increased risk of mortality during the pandemic period was consistent across all subgroups. In both the crude and adjusted models, no significant interaction was found between the pandemic period and any of the subgroup categories, indicating that the effect of the pandemic period on colorectal cancer mortality risk was not significantly modified by these demographic and clinical factors.

## 4 DISCUSSION

This large cohort study showed that the risk of death from colorectal cancer diagnosed during the COVID‐19 pandemic was approximately 1.4 times higher than the risk of death in the pre‐pandemic period. These results were consistent after adjustment for age, sex, stage, and treatment status categories, as well as the results of two sensitivity analyses. The adjusted HRs were smaller than the crude HRs, but the impact of the COVID‐19 pandemic on colorectal cancer mortality was still significant.

In this study, we used a large cancer registry to document an increased mortality risk from colorectal cancer during the COVID‐19 pandemic. The 5‐year survival rate for colorectal cancer among patients diagnosed in 2009 and 2011 showed an improving trend from 69% to 71% in Japan.^18^ A previous multicenter observational study found no change in the mortality risk from gastrointestinal cancer in older adults^15^; however, the study had limited statistical power due to the small sample size. In addition, a national database study of surgical cases in Italy showed that diagnosis at an advanced stage increased after the pandemic,^23^ although the mortality risk was not reported. In this study, we assessed patients diagnosed with colorectal cancer from 2018 to 2021 and conducted a 1 year follow‐up of more than 5000 cases of colorectal cancer treated by endoscopy, laparoscopy, open surgery, chemotherapy, radiation therapy, and endocrine therapy to determine the impact of the COVID‐19 pandemic on the risk of mortality. This study suggests that the impact of the COVID‐19 pandemic on cancer management may persist in 2020 and 2021.

In this study, the risk of death was higher during the pandemic period, even after adjusting for known post‐diagnostic risk factors. Previous studies have identified the following post‐diagnostic risk factors: (1) advanced cancer at diagnosis, (2) a longer interval between diagnosis and starting treatment, and (3) changes in treatment options after diagnosis. A study using Italian National databases found an increased adjusted odds ratio of 1.07 for the risk of death in patients with advanced cancer stage at diagnosis.^23^ In this study, the proportion of Stage 4 cancers increased from 13% to 14%. Regarding the interval between diagnosis and starting treatment, the results of a meta‐analysis showed that a delay of more than 4 weeks between surgery to postoperative chemotherapy and radiation therapy increases the HRs for risk of death to 1.0–1.3.^24^ In this study, we compared the interval from the first visit to the start of each treatment and the interval between treatments in the pandemic and pre‐pandemic periods. The median (IQR) time from diagnosis to treatment was 30 (19–49) days for laparoscopic treatment and 22 (10–38) days for surgical treatment. Contrary to what might be assumed, the interval from diagnosis to laparoscopic surgery and chemotherapy was shorter during the COVID‐19 pandemic period, unlike that in previous studies. This is likely to be due to factors such as a decrease in the overall number of hospital visits following the start of the COVID‐19 pandemic and a decrease in the waiting time before surgery.^11^, ^25^ The number of beds in the acute care wards occupied by patients with COVID‐19 remained below 5%.^26^ Regarding the changes in treatment options, there was a decrease in open surgery and an increase in laparoscopic surgery during the pandemic period, which might be due to the results of a randomized control trial in Japan reported in 2016, showing non‐inferiority of laparoscopic surgery to open surgery.^27^, ^28^ In other aspects, the percentage of endoscopic, laparoscopic, or open surgery for curative treatment was 88% during the pandemic period compared with 91% during the pre‐pandemic period, and a 3% decrease in the frequency of chemotherapy, which may have influenced the prognosis of colorectal cancer. Although the impact of the above risk factors on the prognosis of colorectal cancer is unknown, the fact that the adjusted HR was lower than the crude HR suggests that these changes had some impact on the poorer prognosis of the disease during the pandemic period.

On the contrary, there are several factors not considered nor adjusted in this study including the time between cancer screening and medical examination, the direct impact of COVID‐19, the indirect effect of COVID‐19 pandemic, and changes in other risk factors, such as socioeconomic status (SES), comorbidities, and physical activities, smoking habits, and performance status.^29^, ^30^ Regarding the time between cancer screening and medical examination, a previous study reported that the HR for death was 1.13 for the group with 13–15 months from fecal occult blood test to endoscopy compared to the group with 1–3 months.^31^, ^32^ Regarding the direct impact of COVID‐19, cancer is a known risk factor for COVID‐19 death.^33^ Figure S1A shows the relationship between the period covered by this study and COVID‐19 outbreaks in Hiroshima Prefecture.^26^ The cumulative incidence of COVID‐19 in Hiroshima Prefecture was 0.8% (22,221/2,810,000) by the end of 2021, with a total of 202 deaths, so the increased mortality in patients with colorectal cancer cannot be attributed to the direct effects of COVID‐19 infections. In addition, several case reports have suggested that the immune response to COVID‐19 might suppress cancer progression.^34^, ^35^ Regarding the indirect effects of COVID‐19 and SES as potential risk factors, emerging evidence shows the impact of persistent symptoms following acute illness (“Long COVID”), and the psychological and financial impact of COVID‐19‐related isolation and social distancing on the health outcomes.^36^, ^37^ We also lack the details of comorbidities, performance status, and physical activities, which are important prognostic factors of colorectal cancer. During the pandemic of COVID‐19, these factors might impact the prognosis of colorectal cancers; therefore, further studies that consider these potential risk factors are necessary to better understand the cause of increased mortality among patients with colorectal cancer.

This study has several strengths. We used a general practice‐based database to cover all cases of colorectal cancer diagnosed at hospitals in Hiroshima Prefecture. This allowed us to analyze a large population at risk and to perform a survival analysis for the most important outcome, death. In addition, we set a 1‐year follow‐up period. Despite the shorter follow‐up period, our study showed a significant increase in mortality risk for colorectal cancer during the pandemic period compared with the pre‐pandemic period. This highlights the importance of conducting timely and efficient studies during pandemics to inform clinical practice and improve patient outcomes. Our study is unique in that it includes an analysis of endoscopic treatment and uses a large dataset to demonstrate the negative impact of the pandemic on the outcome of colorectal cancer, which has not been previously explored. This finding adds to the literature on the impact of the COVID‐19 pandemic on cancer outcomes.

This study also has some limitations. First, there is a possibility of selection bias occurring in the groups that were followed up and those that were not (Table S4). The follow‐up group had fewer patients with Stage 3 and an unknown stage and more patients with Stages 0, 1, 2, and 4 than the non‐follow‐up group. The follow‐up group also had a higher frequency of cases that were not treated. We cannot rule out the possibility that the risk of death during the pandemic period was overestimated due to this selection bias. In the registry, it is mandatory to report all the patients diagnosed as colorectal cancer to maintain its role of cancer‐designated hospital, however, whether to report the follow‐up periods for each patient depends on the system of each hospital, not on the patients (Table S5). The proportion of traceable patients depends on the hospital, and the proportion of traceable patients in each hospital did not change markedly between the pre‐pandemic and pandemic period; therefore, serious selection bias would not have occurred. Another limitation is that the outcome of the study was all‐cause mortality; therefore, we were unable to assess cancer‐related mortality specifically. Finally, as we described above, we did not include other risk factors which are important prognostic factors for cancer outcomes, in the adjusted model.

To the best of our knowledge, this study represents the first comprehensive evaluation of the influence of the COVID‐19 pandemic on the survival outcomes of patients with colorectal cancer, encompassing endoscopic treatment, and utilizing a large dataset. The COVID‐19 pandemic was associated with increased mortality risk throughout 2020 and 2021. It is important to monitor cancer outcomes during and after the pandemic, and to identify possible disparities that may contribute to the increased mortality risk. This study did not identify all factors contributing to the mortality risk of colorectal cancer during the COVID‐19 pandemic; therefore, further studies are warranted to better understand the impact of COVID‐19 on cancer outcomes, and the system of care should be adapted to improve the outcomes of cancers worsened by the COVID‐19 pandemic.

## AUTHOR CONTRIBUTIONS

**Daisuke Miyamori:** Conceptualization (lead); data curation (lead); formal analysis (lead); funding acquisition (lead); investigation (lead); methodology (equal); project administration (equal); resources (equal); writing – original draft (lead). **Tsukasa Kamitani:** Methodology (equal); supervision (equal); writing – review and editing (equal). **Shuhei Yoshida:** Methodology (equal); validation (equal). **Yuya Shigenobu:** Validation (equal). **Kotaro Ikeda:** Validation (equal). **Yuka Kikuchi:** Validation (equal). **Saori Kashima:** Investigation (equal); methodology (equal); software (equal). **Yosuke Yamamoto:** Supervision (equal); writing – review and editing (equal).

## FUNDING INFORMATION

This study received support from JSPS KAKENHI Grant Number JP21K17227.

## CONFLICT OF INTEREST STATEMENT

The authors declare no conflicts of interest related to this research. The authors of this study fulfill the authorship criteria set by the International Committee of Medical Journal Editors (ICMJE).

## ETHICS STATEMENT

The study protocol was approved from the Ethical Committee for Epidemiology of Hiroshima University (E2022‐0139) and was implemented according to the principles as outlined in the Declaration of Helsinki. Informed consent was waived because the authors received the data as anonymous status from the registry system.

## Supporting information


Figure S1:

Figure S2:

Figure S3:

Tables S1–S5.

## Data Availability

The data and materials generated and analyzed in this study are not publicly available due to legal restrictions. However, interested researchers may request access to the data from the corresponding author, DM, upon reasonable request.
