# Supplementary figures and images for: Impact of the COVID‐19 pandemic on the mortality among patients with colorectal cancer in Hiroshima, Japan: A large cancer registry study
Source: Cancer Med. 2023 Oct 25;12(21):20554–63. doi: 10.1002/cam4.6630 (PMC10660096; doi:10.1002/cam4.6630)

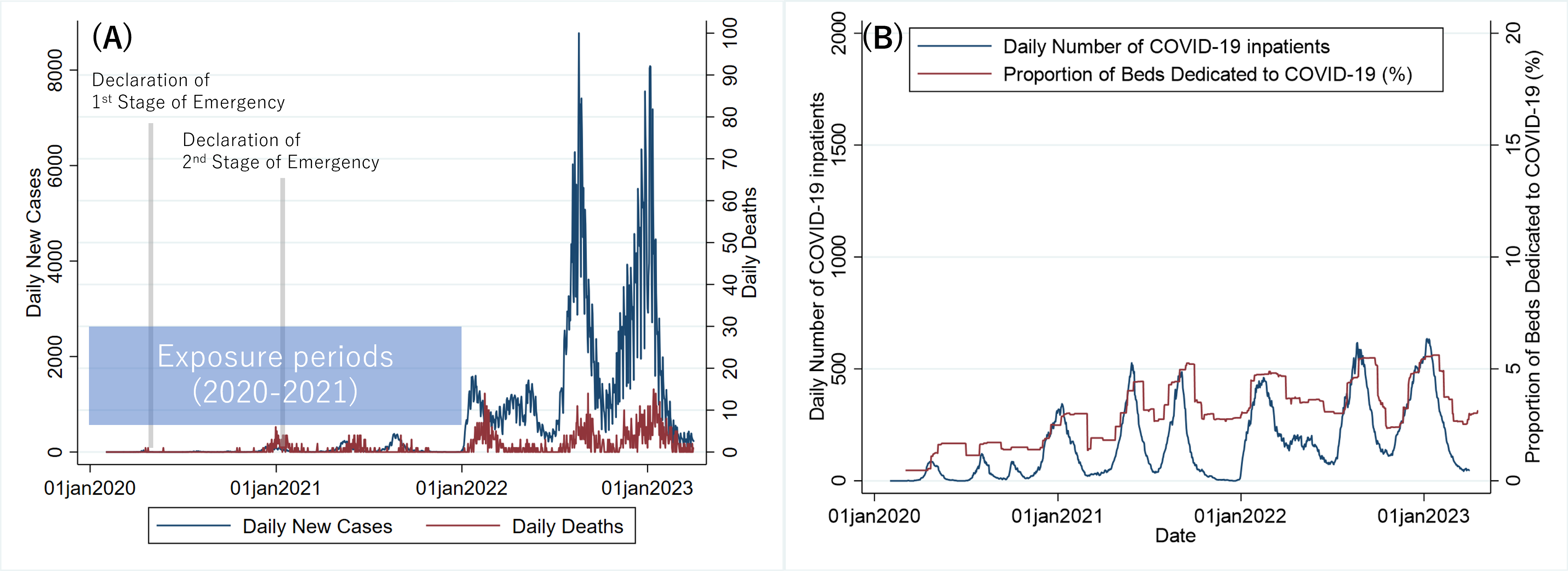

Supplement: Supplementary file 1 — Figure S1: [file CAM4-12-20554-s002.tif]

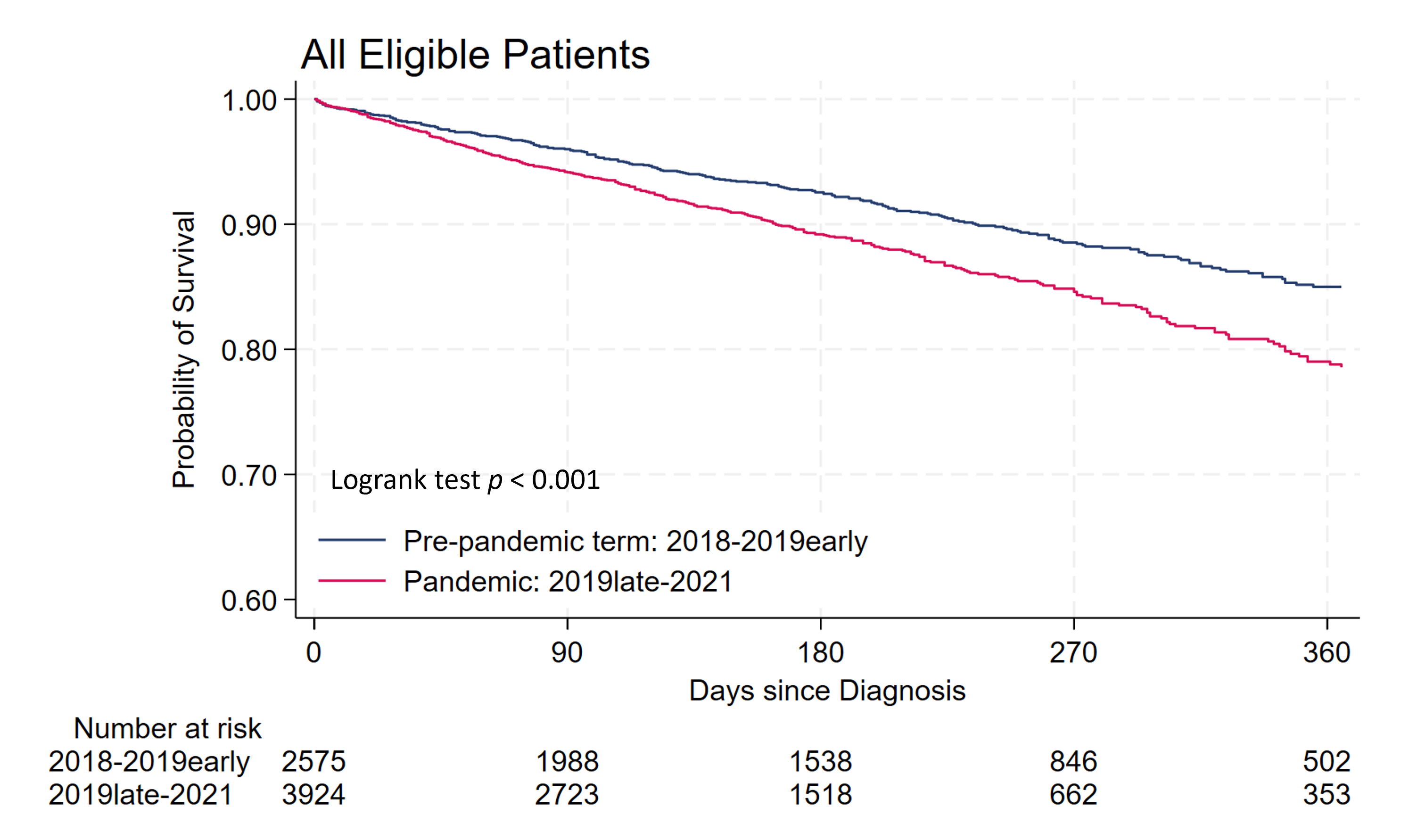

Supplement: Supplementary file 2 — Figure S2: [file CAM4-12-20554-s003.tif]

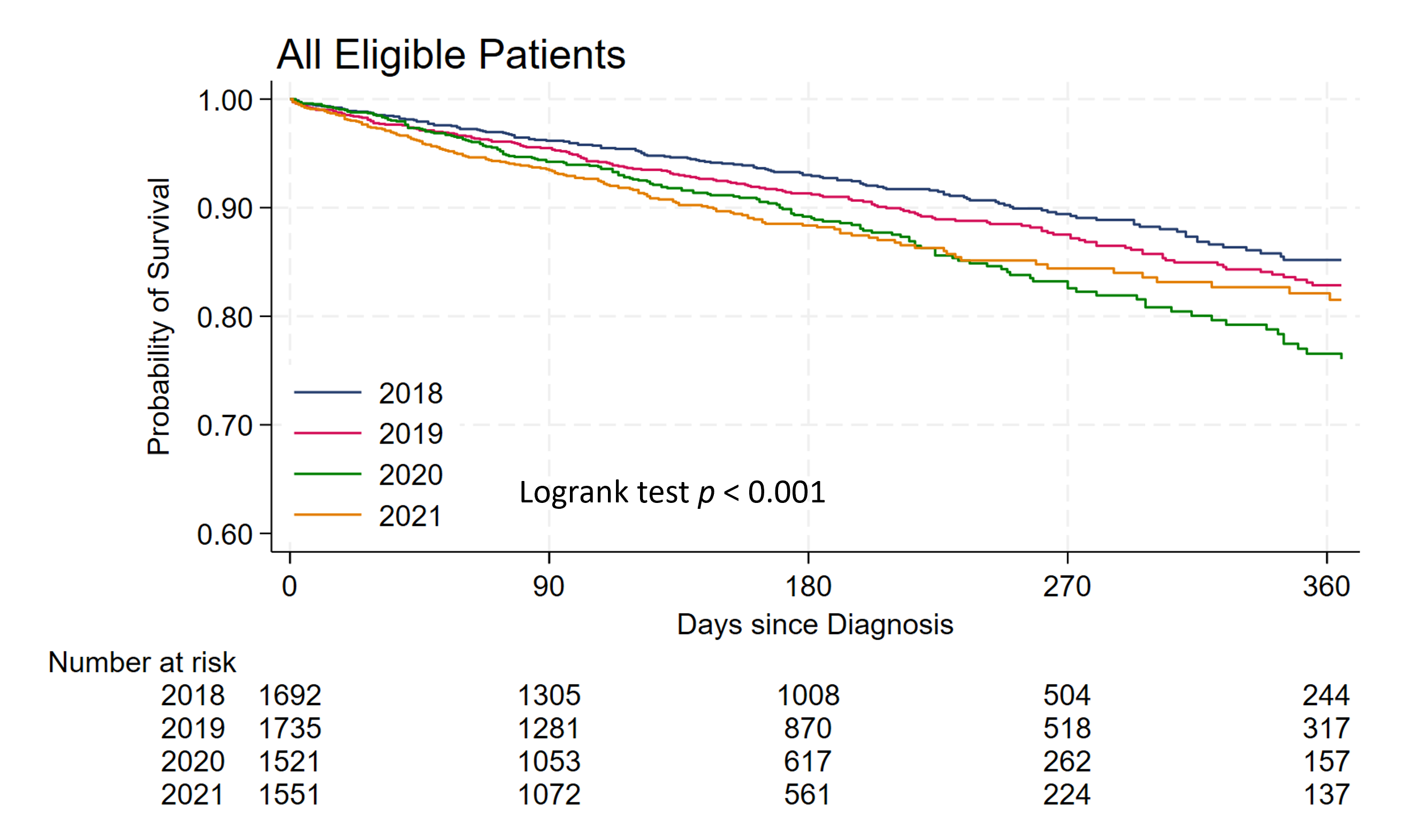

Supplement: Supplementary file 3 — Figure S3: [file CAM4-12-20554-s001.tif]
