# Supplementary material for: Impact of the COVID‐19 pandemic on the mortality among patients with colorectal cancer in Hiroshima, Japan: A large cancer registry study
Source: Cancer Med. 2023 Oct 25;12(21):20554–63. doi: 10.1002/cam4.6630 (PMC10660096; doi:10.1002/cam4.6630)
Supplement: Supplementary file 4 — Tables S1–S5. [file CAM4-12-20554-s004.docx]

Supplementary Table1 ICD-O-3 site Codes included in this study

| ICD-O-3 Site Codes | Site |
| --- | --- |
| C18.0 | Cecum |
| C18.2 | Ascending colon; Right colon |
| C18.3 | Hepatic flexure of colon |
| C18.4 | Transverse colon |
| C18.5 | Splenic flexure of colon |
| C18.6 | Descending colon; Left colon |
| C18.7 | Sigmoid colon |
| C18.8 | Overlapping lesion of colon |
| C18.9 | Colon, NOS |
| C19.9 | Rectosigmoid junction |
| C20.9 | Rectum, NOS |

NOS, not otherwise specified

Supplementary Table2 Additional Characteristics of Patients with Colorectal Cancer: Detection Process, Location, Histology, Tumor Grade, Time from Diagnosis, and Interval between Surgery and Other Treatment

|  | Total | Pre-pandemic | Pandemic |
| --- | --- | --- | --- |
|  | N=6,499 | N=3,427 | N=3,072 |
| Process of Detection, N (%) |  |  |  |
| Screening | 1,412 (22%) | 702 (20%) | 710 (23%) |
| Endoscopic follow-up | 2,346 (36%) | 1,237 (36%) | 1,109 (36%) |
| Autopsy | 3 (0%) | 2 (0%) | 1 (0%) |
| Symptomatic | 2,670 (41%) | 1,452 (42%) | 1,218 (40%) |
| Others | 68 (1%) | 34 (1%) | 34 (1%) |
| Location, N (%) |  |  |  |
| Cecum | 558 (9%) | 279 (8%) | 279 (9%) |
| Ascending colon; Right colon | 1,064 (16%) | 584 (17%) | 480 (16%) |
| Hepatic flexure of colon | 113 (2%) | 54 (2%) | 59 (2%) |
| Transverse colon | 620 (10%) | 331 (10%) | 289 (9%) |
| Splenic flexure of colon | 51 (1%) | 23 (1%) | 28 (1%) |
| Descending colon; Left colon | 316 (5%) | 179 (5%) | 137 (4%) |
| Sigmoid colon | 1,743 (27%) | 919 (27%) | 824 (27%) |
| Colon, NOS | 638 (10%) | 331 (10%) | 307 (10%) |
| Rectosigmoid junction | 1,386 (21%) | 721 (21%) | 665 (22%) |
| Rectum, NOS | 558 (9%) | 279 (8%) | 279 (9%) |
| Histology |  |  |  |
| Adenocarcinoma | 6,100 (94%) | 3,199 (93%) | 2,901 (94%) |
| Epithelial carcinoma | 42 (1%) | 30 (1%) | 12 (0%) |
| Cystic, mucinous and serous carcinoma | 156 (2%) | 101 (3%) | 55 (2%) |
| Hodgkin and non-Hodgkin Lymphoma | 35 (0%) | 17 (0%) | 18 (0%) |
| Others | 13 (0%) | 5 (0%) | 8 (0%) |
| Neoplasm, NOS | 153 (2%) | 75 (2%) | 78 (3%) |
| Tumor Grade |  |  |  |
| Well-differentiated | 3,698 (57%) | 1,861 (54%) | 1,837 (60%) |
| Moderate-differentiated | 2,084 (32%) | 1,140 (33%) | 944 (31%) |
| Poorly-differentiated | 227 (3%) | 125 (4%) | 102 (3%) |
| Undifferentiated | 1 (0%) | 0 (0%) | 1 (0%) |
| Unknown | 31 (0%) | 16 (0%) | 15 (0%) |
| Time between surgery and other treatment (days), median (IQR) |  |  |  |
| Chemo to open | 38 (32-48) | 39 (32-48) | 36 (28-48) |
| Radiation to open | 18 (13-22) | 22 (22-22) | 13 (13-13) |
| Open to radiation | 92 (88-99) | 90 (82-94) | 96 (88-102) |
| Open to chemo | 115 (92-145) | 122 (92-152) | 102 (96-129) |
| Chemo to laparoscopy | 38 (31-48) | 39 (30-48) | 37 (31-48) |
| Radiation to laparoscopy | 5 (3-7) | 7 (7-7) | 3 (3-3) |
| Laparoscopy to radiation | 96 (90-100) | 93 (87-99) | 98 (90-103) |
| Laparoscopy to chemo | 99 (91-108) | 95 (86-106) | 101 (96-118) |

Location were classified based on ICD-O-3 site code. Histology and Tumor Grade were also classified based on ICD-O-3 code. NOS: not otherwise specified, N/A: not applicable. IQR: interquartile range, N: number.

Supplementary Table3 Baseline Characteristics of Enrolled Patients

|  | Total | Pre-pandemic term | Pandemic term |
| --- | --- | --- | --- |
|  | N=15,085 | N=7,843 | N=7,242 |
| Age, Median (IQR)-yr | 72 (65-80) | 72 (65-79) | 73 (65-80) |
| Sex (male), No (%) | 8,939 (59%) | 4,685 (60%) | 4,254 (59%) |
| Cancer stage, No (%) |  |  |  |
| 0 | 3,008 (20%) | 1,593 (20%) | 1,415 (20%) |
| 1 | 3,067 (20%) | 1,551 (20%) | 1,516 (21%) |
| 2 | 2,459 (16%) | 1,244 (16%) | 1,215 (17%) |
| 3 | 2,031 (13%) | 1,073 (14%) | 958 (13%) |
| 4 | 1,888 (13%) | 947 (12%) | 941 (13%) |
| Unknown | 2,624 (17%) | 1,427 (18%) | 1,197 (17%) |
| Missing | 8 (0%) | 8 (0%) | 0 (0%) |
| Treatment status, No (%) |  |  |  |
| Endoscopic treatment | 6,255 (41%) | 3,292 (42%) | 2,963 (41%) |
| Laparoscopic surgery | 5,489 (36%) | 2,745 (35%) | 2,744 (38%) |
| Open surgery | 2,053 (14%) | 1,190 (15%) | 863 (12%) |
| Radiotherapy | 365 (2%) | 194 (2%) | 171 (2%) |
| Chemotherapy | 3,253 (22%) | 1,726 (22%) | 1,527 (21%) |
| Endocrine therapy | 5 (0%) | 2 (0%) | 3 (0%) |
| No treatment recorded | 1,206 (8%) | 607 (8%) | 599 (8%) |

Cancer stage is classified into 5 levels according to the 8^th^ edition of the Union for International Cancer Control Tumor Node Metastasis classification, ; IQR, interquartile range; No., number

Supplementary table4 Comparison by follow-up status

|  |  | Total | No follow-up | Follow up |
| --- | --- | --- | --- | --- |
|  |  | N=15,085  Pre7843/Post7242 | N=8,583  Pre4413/Post4170 | N=6,502  Pre3430/Post3072 |
| Age, Median (IQR) |  | 72 (65-80) | 73 (66-80) | 72 (64-79) |
| Male sex, No (%) |  | 8,939 (59%) | 5,110 (60%) | 3,829 (59%) |
| Cancer stage, No. (%) | 0 | 3,008 (20%) | 1,566 (18%) | 1,442 (22%) |
|  | 1 | 3,067 (20%) | 1,637 (19%) | 1,430 (22%) |
|  | 2 | 2,459 (16%) | 1,378 (16%) | 1,081 (17%) |
|  | 3 | 2,031 (13%) | 1,298 (15%) | 733 (11%) |
|  | 4 | 1,888 (13%) | 951 (11%) | 937 (14%) |
|  | Unknown | 2,624 (17%) | 1,748 (20%) | 876 (13%) |
|  | Missing | 8 (0%) | 5 (0%) | 3 (0%) |
| Treatment status, No. (%) | Endoscopic treatment | 6,255 (41%) | 3,685 (43%) | 2,570 (40%) |
|  | Laparoscopic surgery | 5,489 (36%) | 3,137 (37%) | 2,352 (36%) |
|  | Open surgery | 2,053 (14%) | 1,162 (14%) | 891 (14%) |
|  | Radiotherapy | 365 (2%) | 213 (2%) | 152 (2%) |
|  | Chemotherapy | 3,253 (22%) | 1,824 (21%) | 1,429 (22%) |
|  | Endocrine therapy | 5 (0%) | 4 (0%) | 1 (0%) |
|  | No treatment recorded | 1,206 (8%) | 599 (7%) | 607 (9%) |

Cancer stage is classified into 5 levels according to the 8^th^ edition of the Union for International Cancer Control Tumor Node Metastasis classification, ; IQR, interquartile range; No., number

Supplementary table5 The number and proportion of traceable patients in each hospital

|  | Pre-pandemic (N=7843) | |  | Pandemic (N=7242) | |  |
| --- | --- | --- | --- | --- | --- | --- |
| Hospital | Presence of follow-up | | Proportion of follow-up (%) | Presence of follow-up | | Proportion of follow-up (%) |
|  | Yes | No |  | Yes | No |  |
| A | 718 | 0 | 100 | 625 | 4 | 99.4 |
| B | 494 | 0 | 100 | 454 | 2 | 99.6 |
| C | 340 | 0 | 100 | 318 | 0 | 100 |
| D | 772 | 1 | 99.9 | 758 | 0 | 100 |
| E | 725 | 7 | 99 | 561 | 6 | 98.9 |
| F | 105 | 372 | 22 | 86 | 353 | 19.6 |
| G | 82 | 326 | 20.1 | 65 | 316 | 17.1 |
| H | 29 | 207 | 12.3 | 34 | 219 | 13.4 |
| I | 17 | 132 | 11.4 | 11 | 124 | 8.1 |
| J | 46 | 494 | 8.5 | 56 | 536 | 9.5 |
| K | 23 | 256 | 8.2 | 13 | 247 | 5 |
| L | 34 | 552 | 5.8 | 43 | 572 | 7 |
| M | 15 | 473 | 3.1 | 24 | 402 | 5.6 |
| N | 26 | 1,183 | 2.2 | 12 | 939 | 1.3 |
| O | 4 | 410 | 1 | 12 | 450 | 2.6 |
| Capital letter (A-O) indicate cancer designated hospital. N, number. | | | | | | |
